# The resilience of learners with specific learning disability in unequally resourced learners with special education needs schools in diverse contexts

**DOI:** 10.4102/ajod.v11i0.1044

**Published:** 2022-11-29

**Authors:** Daphney Mawila

**Affiliations:** 1 Department of Educational Psychology, Faculty of Education, University of Johannesburg, Johannesburg, South Africa

**Keywords:** learners with educational special needs, less resourced, resilience, resilience enabling, specific learning disability, social-ecological, well resourced, unequal opportunities

## Abstract

**Background:**

Despite the inequality of resources in South Africa, learners in less-resourced schools and contexts can be resilient in the face of adversities.

**Objectives:**

This study sought to investigate the impact of unequal resources in diverse contexts and schools on the resilience of learners with specific learning disability (SLD) in learners with special education needs (LSEN) schools in South Africa.

**Method:**

A quantitative explanatory design was adopted and respondents were selected using a purposive sampling technique. A sample of 217 learners with SLDs across four LSEN schools located in diverse contexts in the Gauteng province, was selected. Data were collected using the Child and Youth Resilience Measure (CYRM-28). The SPSS software was used to analyse the data and one-way analysis of variance was used as a statistical technique.

**Results:**

The results showed that resilience scores did not yield a significant statistical difference among learners from unequally resourced schools (*p* = 0.300 > 0.05) and diverse contexts (*p* = 0.173 > 0.05). These results suggest that resilience was the same across unequally resourced schools and diverse contexts; thus, all learners are capable of resilience regardless of these contexts.

**Conclusion:**

Resilience of learners with SLD was not necessarily associated with the accessibility of resources in their contexts but with their agency in identifying them and using them meaningfully to combat their learning disabilities.

**Contribution:**

The study contributes to the limited body of knowledge on the resilience of learners with SLD in unequally resourced contexts and LSEN schools.

## Introduction

An increasing concern about what makes individuals cope with adversity in diverse contexts is growing globally. Prior resilience studies (Masten 2014; Theron 2016; Van Breda 2018; Van Rensburg, Theron & Ungar 2019) primarily focused on the socio-ecological processes and resources that predict resilience in individuals exposed to hardships. Ungar (2011) stated that building available and accessible physical and social resources for individuals enables their resilience to cope well with severe stressors. Zautra, Hall and Murray (2010) affirmed that development, recovery and sustainability are probable and are extremely reliant on the resources available in a specific context. In alignment with this, resilience is defined as an individual’s capability to bounce back from hardship and their ability to access available resources in their context to sustain and enhance well-being and the capability of communities to avail meaningful resources (Ungar 2011). Thus, resilience depends on the individual’s ability to navigate their environments and access resources to foster their resilience when faced with risk factors.

Equity of resilience resources in South African schools and communities is a matter of concern; some schools, especially in most urban areas, are well resourced while others in rural areas and townships are less resourced. Some studies (e.g. Theron 2016; Van Breda & Theron 2018) showed that well-resourced schools and contexts are more resilience-enabling compared with the less-resourced settings. A study by Johnson and Lazarus (2008) showed that resourced schools and contexts have fewer students presenting with risky behaviours. The authors add that these schools have sufficient support structures to build students’ resilience despite adversity. Among South African youth in less-resourced schools and contexts, adversities are common and prominent (Van Breda & Theron 2018). Dickens (2018) described that South Africans face several socio-economic challenges, such as unemployment and poverty, which result in millions of students struggling to complete their studies and get employment. A study by Theron (2016) stated that a black woman raised in single-parenting families in a poor context with limited resources is likely to receive inferior schooling and poor support from caregivers and experience violence, which will complicate her life path. Theron added that these adversities and co-occurring risks will probably lead to a lack of opportunities to defeat the odds that work against her, especially if socio-ecological stakeholders do not intervene. Moreover, the country’s socio-economic profile hinders South African learners’ capability to effectively deal with the adversities they face (Van Breda & Theron 2018).

Theron and Theron (2010) stipulated that a supportive and safe school environment tends to buffer the effect of risks by providing protective factors and promoting resilience for its learners. As most of the schools in rural areas and townships have limited resources, *are they able to support or develop the resilience of their learners, considering the challenges and their troublesome environments?* In adverse environments such as those where these schools are located, Van Breda and Theron (2018) questioned how some learners demonstrate better-than-expected competency and do well. Contrary to expectations, the study on South African youth resilience by Van Breda (2017a) asserted that the highest levels of resilience were found in children’s homes in poorer communities. This study argues that everyone, whether in resourced or less-resourced schools and contexts, is capable of resilience. Resilience is enabled by the individuals’ capacity to access and utilise coping resources in their environment. Van Breda (2017a) found that young people’s resilience was not associated with the accessibility of resources in their social context but with their agency to recognise these resources as prospects to mobilise them to cope with unfavourable circumstances. This study supports the notion that every learner is capable of resilience regardless of unequal resources presented to them at school and within their contexts. Limited research has been conducted on resilience and unequal resources in different contexts and learners with special education needs (LSEN) schools. Thus, the novelty of this study lies in its intention to investigate whether unequal opportunities in diverse contexts and LSEN schools influence the resilience of learners with specific learning disability (SLD). The study sought to add to the growing body of knowledge regarding the resilience of learners with SLD in diverse schools and contexts. Furthermore, the study could offer insights into the support needs of SLD learners’ resilience development in LSEN schools.

### Specific learning disability

Specific learning disability is defined by the American Psychiatric Association (2013:32) as a neurodevelopmental disorder ‘diagnosed when there are specific deficits in an individual’s ability to perceive or process information efficiently and accurately’. Specific learning disability’s origins can be traced to biology and the interaction between genetics and environmental factors. American Psychiatric Association (2013) stated that this interaction negatively impacts an individual’s brain capacity to perceive and process information efficiently. Gow, Mostert and Dreyer (2020) stated that an individual’s basic cognitive processing in written and spoken language is negatively affected by SLD. Bandla, Mandadi and Bhogaraju (2017) indicated that the manifestation of SLD occurs during the individual’s early years of formal education and results in challenges in learning basic foundational scholastic skills such as writing (dysgraphia), mathematics (dyscalculia) and reading (dyslexia) and co-exists. Thus, these difficulties have a devastating long-term impact on the individual’s capacity to function daily, especially in tasks that include written words, mastery of numbers, written expression and reading (American Psychiatric Association 2013). As a result of these difficulties, an SLD is considered a risk factor for individuals’ development. Similarly, Harðardóttir, Júlíusdóttir and Guðmundsson (2015) pointed out that SLD is regarded as a risk factor that predicts adverse outcomes. Although SLD is adversity, resources within their social ecology can promote their resilience, which could be used to conquer the challenges that accompany the presence of SLD (Lance et al. 2015). Venkatesan (2017) reported that the diagnosis of SLD is not as simple as noting the list of signs and symptoms an individual presents with. Several factors, including academic achievement level and cognitive, sensory, adaptive, social and emotional functioning, should be considered before diagnosing an individual with SLD. In multilingual and cultural contexts such as South Africa, the diagnosis of SLD is complex because several assessment tools used to assess SLD were not developed for South African populations and lack appropriate norms. A study conducted in India by Kohli, Sharma and Padhy (2018) revealed that various batteries used to assess SLDs lacked well-established norms and are based on a limited sample, making diagnosing individuals with SLD difficult. In South Africa, where a high number of learners are first-generation learners with limited home support, the limited infrastructure, poorly trained teachers, inadequate teaching aids and controversies on learning in a second and third language should also be considered before making such a diagnosis. The respondents of this study were already diagnosed with SLD and were in LSEN schools. Based on the aforementioned diagnosis difficulties, the author acknowledges that there is a possibility that the respondents of this study may have been misdiagnosed with SLD; however, the scope of this study is not investigating the appropriateness of their diagnosis but the resilience of learners with SLD in diverse schools and contexts.

### Socio-ecological framework of resilience

This study employed insights from the socio-ecological framework of resilience. Ungar (2011) stipulated that this framework traces its origin to the ecological system theory proposed by Bronfenbrenner in 1979. For decades, the socio-ecological framework of resilience gained popularity and it has been used to understand the reasons as to why certain people resile from adversity whereas others do not (Van Breda 2017b). The socio-ecological framework of resilience is defined as ‘the ability of individuals, families and communities to navigate and seek out meaningful social and ecological resources that provide protective factors in times of stress’ (Höltge et al. 2020:19). Theron (2018) posited that this framework emphasises individuals’ social ecologies in enhancing and promoting their resilience. Within this framework, Ungar (2011) stated that resilience is:

[*T*]he child’s ability to navigate their way to social, psychological, and physical resources that sustain their well-being amid adversity and their ability to negotiate for these resources to be provided to them in culturally meaningful ways. (p. 225)

In addition, Wang, Liu and Qi (2014) asserted that multiple factors (such as individual, relationships and contextual) influence the resilience development process, thus making the concept of resilience complex. Aldwin and Igarashi (2012) stated that these factors interrelate at the individual, environmental and socio-cultural levels. The levels provide resources for an individual’s resilience. Studies (e.g. Masten 2014; Ungar 2013) have pointed out that socio-ecological stakeholders, such as caregivers, practitioners, community leaders, policymakers and service providers, are jointly responsible for individuals’ attainment of desirable outcomes. A study by Theron (2016) contended that the stakeholders need to avail the resources necessary for individuals with unfavourable life circumstances. In this way they can help individuals combat negative life outcomes in different contexts.

Even though South Africa’s contextual challenges hamper the capacity of individuals to deal effectively with challenges (Van Breda & Theron 2018), resilience research demonstrates that individuals overcome hardships and become competent, resilient and successful individuals (Malindi 2014; Ungar 2011). This applies to learners in less-resourced schools and contexts as every community has protective factors that enable individuals to cope with adversity. It is, thus, the individual’s duty to identify and utilise these factors to overcome hardships. Stakeholders (such as caregivers, schools, teachers and community members) must capacitate learners in navigating their respective contexts and search for resilience-enabling resources.

This study intended to investigate the impact of unequal resources in diverse contexts and schools on the resilience of learners with SLD in LSEN schools in South Africa. The specific research question was as follows: *Do unequal resources in diverse LSEN schools impact learners’ resilience with SLD?* The findings would effectively reveal that all learners with SLD can develop resilience irrespective of the unequal resources in their environment. The insights of this study would also assist stakeholders in realising that limited resources should not hinder them from enabling the resilience of learners with SLD.

The formulated hypotheses for this study are stated as follows:

**H**_**0**_: There is no significant difference in resilience across different contexts and schools with unequal resources.

**H**_**1**_: There is a significant difference in resilience across different contexts and schools with unequal resources.

## Research methods and design

### Approach and design

An exploratory quantitative research design was employed in this study as it permits comparisons among learners in LSEN schools and diverse contexts. This research is exploratory because it attempted to investigate the less researched area. Data were collected over a limited time, making this study cross-sectional.

### Setting

The study participants were selected from four LSEN schools located in Johannesburg North, Soweto, West Rand and Elspark. Compared with the three schools, the LSEN school in Soweto is situated in a township where most residents face socio-economic challenges, with contextual adversities and elevated unemployment levels. The residents in this context are black African individuals, previously disadvantaged and fall within a low socio-economic status. Therefore, the school in Soweto has restricting infrastructures and few resources. Services such as occupational, psychological and remedial services are not offered at the school. Psychological services are sought from the Gauteng Department of Education (GDE) experts, who are not always readily available because of numerous schools needing services in this specific district.

The LSEN schools in Johannesburg North, West Rand and Elspark are located in urban areas. Most of the inhabitants are in the middle to upper socio-economic status. Unlike the LSEN school in Soweto, learners pay school fees in these contexts. As these are public schools, the exemption policy applies to parents who cannot afford the school fees. Parents must apply to the schools to receive an exemption from paying school fees. As a result of the limited LSEN schools for learners with SLD across the Gauteng province in South Africa, learners are not limited to attending schools close to their homes as stipulated in the guidelines for public mainstream schools. In these urban schools, few numbers of learners in a class permit specialised teaching by competent teachers and individualised attention. These schools have adequate infrastructure and various supportive resources, including occupational and speech therapists, remedial support, social workers and learning support experts, psychologists and nurses. This study comprised a sample of 69 learners from Elspark, 96 learners from the West Rand, 42 learners from Soweto and 10 learners from Johannesburg North.

### Study population and sampling strategy

Learners presenting with SLD were purposefully selected to partake in this study because they were best suited for the purpose of the study (Townsend & De la Rey 2016). In line with the focus of the study, the sampling criteria included learners already diagnosed with SLD and who were placed in LSEN schools. Learners were selected based on their interest in participating in this study and participants were drawn from four LSEN combined schools. Combined schools cater for both primary and secondary school-going learners. The sample consisted of 217 learners with SLD in LSEN schools, boys and girls aged between 9 and 19 years.

### Research tool

The Child and Youth Resilience Measure (CYRM-28) was used to collect quantitative data. The CYRM-28 is an existing questionnaire, which has been used across cultures and countries (e.g. Amirsardari et al. 2016; Langham et al. 2018; Sanders et al. 2015; Zand, Liebenberg & Shamloo 2016). In South Africa, studies (e.g. Govender et al. 2017; Van Rensburg, Theron & Ungar 2017) also found that CYRM-28 is a reliable and valid measure of resilience. ‘CRYM-28 seeks to provide a more comprehensive understanding of the processes of resilience across culture and context, accounting for the heterogeneity of culture and experiences of youth’ (Liebenberg et al. 2012:87). The CYRM-28 items provide a reliable and accurate measure of resilience across cultures as it was developed by multicultural research teams across different countries (Ungar & Liebenberg 2011). The CYRM-28 is a self-report paper-based measure comprising 28 items on a five-point Likert scale (1 = not at all, 5 = a lot). It takes 20 min to complete the measure. However, in this study, learners were given more time (40 min) to complete the measure because of the nature of their diagnosis. Three multilingual University of Johannesburg master’s student psychologists were employed as field workers whom the researcher trained. The focus of the study was discussed with them so that they could be familiar with the study. Their role included reading the CYRM-28 to learners and assisting with completing the items. Cronbach’s alpha coefficient was used to analyse the reliability of the CYRM-28. Internal consistency of a measure ranges between 0 and 1, where 0.0 means no consistency and 1.0 is a perfect consistency in measurement (Tavakol & Dennick 2011). In this study, the CYRM-28 had a high internal consistency as the Cronbach’s alpha value was 0.867.

### Data collection

As mentioned in the previous section, three multilingual field workers were employed to assist with data collection. Learners, especially in Soweto, struggled with reading the questionnaire. Some translation was required because of language barriers, which could have impacted the measure’s reliability and results thereof. To ensure a precise understanding of the CYRM-28, items were translated for 42 learners (in the school based in Soweto) as most did not understand English. As a result, data collection was prolonged because of difficulty in translating instructions to African languages. Translations are likely to have compromised the reliability of the CYRM-28. However, as stated earlier, a Cronbach’s alpha of 0.867 suggested that the CYRM-28 was a reliable measure of resilience for learners with SLD. Thus, CYRM-28 appropriately measures the resilience of learners who present with SLD in the Gauteng province, South Africa.

### Data analysis

The Statistical Package for the Social Science (SPSS) software (version 25) was used to analyse the data collected using the CYRM-28. One-way analysis of variance (ANOVA) statistical technique was utilised to investigate whether a statistically significant difference existed across unequal resources in four LSEN schools and contexts. One-way ANOVA compares the means of the study’s sample, and it is an extension of two independent *t*-test samples to more than two groups (Ostertagova & Ostertag 2013).

### Ethical considerations

The University of Johannesburg Ethics Committee at the Faculty of Education granted ethical clearance (Sem 2 2018-007) to conduct this study. The GDE and school headmasters also gave permission to collect data. Parents of learners consented for their children to be part of the study and learners signed informed assent forms.

## Results

### Demographic characteristics

Table 1 illustrates the frequency distribution of the respondent’s demographic characteristics. The sample comprised respondents aged 9–19 years; 153 (70.5%) of the participants were boys and 64 (29.5%) were girls. Most respondents were between the ages of 12 and 14 years, demonstrating 77 (35.5%) of the sample. A total of 76 (35%) respondents were aged between 9 and 11 years and the smallest number of respondents, 16 (7.4%), were between 18 and 19 years of age. The majority of respondents’ home language was English (89; 41.0%), followed by IsiZulu (43; 19.8%) and Setswana (29; 13.4%), while Xitsonga, siSwati, Tshivenda and other (Portuguese) languages had the least number of participants, respectively. The majority of the respondents were in grade 5, signified by 44 (20.3%), followed by 39 (18.0%) in grade 6 and 32 (14.7%) in grade 4. The lowest number of respondents were in grades 12 (1.4%) and 8 (4.1%), respectively.

**TABLE 1:** Demographic characteristics.

Characteristics	*N*	%
**Gender**		
Male	153	70.5
Female	64	29.5
**Age**		
9–11 years	76	35.0
12–14 years	77	35.5
15–17 years	48	22.1
18–19 years	16	7.4
**Language**		
Afrikaans	27	12.4
English	89	41.0
Xitsonga	3	1.4
Southern Sesotho	11	5.1
Northern Sesotho	6	2.8
Setswana	29	13.4
IsiZulu	43	19.8
SiSwati	3	1.4
Xhosa	4	1.8
Tshivenda	1	0.5
Other (Portuguese)	1	0.5
**Grade**		
Grade 3	14	6.5
Grade 4	32	14.7
Grade 5	44	20.3
Grade 6	39	18.0
Grade 7	19	8.8
Grade 8	9	4.1
Grade 9	20	9.2
Grade 10	18	8.3
Grade 11	19	8.8
Grade 12	3	1.4

*Source*: Mawila, D., 2019, ‘Relationship between resilience and social ecological support among learners with specific learning disability in LSEN schools’, PhD thesis, University of Johannesburg, Johannnesburg.

### One-way analysis of variance on the impact of different contexts on resilience

One-way ANOVA was used to examine the impact of different contexts on resilience. Respondents in this study were divided into three contexts: urban, rural and township. As the study focused on learners in diverse contexts with unequal resources, this demarcation was necessary. Table 2 presents the percentage breakdown distribution according to respondents’ residence area.

**TABLE 2:** Area of residence.

Variable	Area of residence			
	Frequency	%	Valid (%)	Cumulative (%)
**Valid**				
Urban area	87	40.1	40.1	40.1
Township	118	54.4	54.4	94.5
Rural area	12	5.5	5.5	100.0

**Total**	**217**	**100.0**	**100.0**	**-**

*Source*: Mawila, D., 2019, ‘Relationship between resilience and social ecological support among learners with specific learning disability in LSEN schools’, PhD thesis, University of Johannesburg, Johannnesburg

Table 2 shows that most of the respondents in this study lived in a township area, representing 118 (54.4%), followed by those in an urban area, described by 87 (40.1%) and lastly, 12 (5.5%) in the rural area. Table 3 presents the results of the impact of different contexts on resilience.

**TABLE 3:** Analysis of variance resilience across different contexts.

Source of variation	ANOVA: Resilience				
	Sum of squares	*df*	Mean square	*F*	Significance
Between groups	724.896	2	362.448	1.769	0.173
Within groups	43846.809	214	204.892	-	-

**Total**	**44571.705**	**216**	**-**	**-**	**-**

*Source*: Mawila, D., 2019, ‘Relationship between resilience and social ecological support among learners with specific learning disability in LSEN schools’, PhD thesis, University of Johannesburg, Johannnesburg

ANOVA, Analysis of variance; *df*, degrees of freedom.

As evident in Table 3, the *p*-value = 0.173 > 0.05 significance level reveals no statistically significant difference in resilience scores among learners with SLD from diverse contexts. Thus,across contexts, resilience is the same. This result suggests that resilience is not reliant on the area where an individual resides and existing in a particular environment does not guarantee an ability to resile.

### One-way analysis of variance on the impact of different schools on resilience

One-way ANOVA was also performed to investigate the impact of different schools on resilience. Table 4 illustrates the frequency distribution of the respondents’ schools.

**TABLE 4:** Distribution of respondents per school.

Variable	Schools			
	Frequency	%	Valid (%)	Cumulative (%)
**Valid**				
Elspark	69	31.8	31.8	31.8
West Rand	96	44.2	44.2	76.0
Soweto	42	19.4	19.4	95.4
Johannesburg North	10	4.6	4.6	100.0

**Total**	**217**	**100.0**	**100.0**	**-**

*Source*: Mawila, D., 2019, ‘Relationship between resilience and social ecological support among learners with specific learning disability in LSEN schools’, PhD thesis, University of Johannesburg, Johannnesburg

Most of the participants were from a school based in West Rand, represented 96 (42.2%) participants, followed by 69 (31.8%) in Elspark and 42 (19.4%) in Soweto. A school in Johannesburg North had the least number of participants, 10 (4.6%). Table 5 demonstrates the study’s results on the impact of different schools on resilience.

**TABLE 5:** Analysis of variance resilience across different schools.

Source of variation	ANOVA: Resilience				
	Sum of squares	*df*	Mean square	*F*	Sig.
Between groups	758.359	3	252.786	1.229	0.300
Within groups	43813.346	213	205.696	-	-

**Total**	**44571.705**	**216**	**-**	**-**	**-**

*Source*: Mawila, D., 2019, ‘Relationship between resilience and social ecological support among learners with specific learning disability in LSEN schools’, PhD thesis, University of Johannesburg, Johannnesburg

ANOVA, Analysis of variance; *df*, degrees of freedom; Sig., Significance.

Table 5 shows no statistically significant difference in resilience scores among learners from different schools, as revealed by the *p*-value = 0.300 > 0.05 significance level. This study found that resilience was the same across different schools. Therefore, resilience was the same across schools in diverse LSEN schools.

The study hypothesised that ‘there is no statistically significant difference in resilience across different contexts and schools with unequal resources’; this hypothesis was therefore accepted.

## Discussion

Learners with SLD face numerous adversities in life; thus, resilience can help them navigate these adversities towards positive life outcomes. Learners in under-resourced contexts and schools have added contextual challenges such as poverty and a lack of or limited resources, making them more vulnerable. The need for resilience resources is highly critical in such contexts. Unlike studies such as Theron (2018) and Van Breda and Theron (2018) that found that well-resourced schools and contexts are linked to greater resilience, the results of this study show that resilience is the same across unequally resourced contexts and LSEN schools. Learners presenting with SLD can develop resilience, regardless of being in a well-resourced or less-resourced environment. The results of this study contest the perception that well-resourced schools and contexts predict higher resilience levels than less-resourced ones. In correlation with this result, researchers such as Mampane (2014), Theron and Theron (2010) and Van Rensburg et al. (2019) have shown that even the poorest communities have resilience resources to draw soothing factors in the face of hardships. Van Breda (2017a) also found that regardless of social contexts, children from impoverished communities can be resilient and their resilience could equal or exceed that of children from better-resourced or wealthier neighbourhoods. Studies focusing on resilience have consistently confirmed that children and youths with unequal resources overcome debilitating adversity in their contexts and develop into resilient individuals (Malindi 2014; Masten 2014; Ungar 2013; Van Breda 2017b; Van Rensburg et al. 2019). In addition, a study by Mampane (2014) showed that township school learners displayed resilience despite the contextual factors that serve as hurdles in their development.

As this study rules out that learners with SLD can develop resilience irrespective of their unequal resource contexts, *what could be attributed to their resilience?* The researcher argues that the capacity of learners with SLD to navigate their unequally resourced contexts and search for socio-emotional, physical and psychological resources that maintain their functioning amid adversity explains this resilience (Ungar 2015). Despite residing in unequal-resourced contexts and LSEN schools, this study contends that learners with SLD should navigate their social ecology to find accessible and meaningful resources. Their resilience is thus entirely reliant on their capability to use the accessible resources to flourish and persist regardless of their SLD. Thus, capacitating learners presenting with SLD to navigate their environments and identify and use accessible resilience resources is imperative. Moreover, Ungar (2006) affirmed that individuals should exercise their agency in navigating pathways towards resilience resources. The results of this study highlight that it is not the number of resources that enable learners with SLD to resile but their ability to draw from available socio-ecological resources within their unequally resourced contexts and LSEN schools. In line with the given discussion on the Social-ecological framework of resilience, navigating and identifying these resources in their unequally resourced contexts and LSEN schools accounts for their resilience. The researcher notes that similar studies have been performed; however, none of the studies has been carried out on LSEN schools and their contexts. The results of this study are unique as it challenges learners with SLD with limited resources not to give in to feelings of helplessness or incapability. This study also acknowledges that learners with SLD need to optimise the little or limited resources in their contexts to enhance their resilience.

## Conclusion

This study investigated the impact of unequal resources on learners’ resilience with SLD in South African LSEN schools and diverse contexts. The results revealed no significant difference in resilience across different contexts and schools with unequal resources. Thus, learners with SLD can develop resilience despite the unequal resilience resources they are presented within their schools and contexts. This study further pointed out that it is not necessarily the quantity of the resilience resources a learner with SLD is presented with, but their ability to navigate their contexts in search of these resources and utilise them for their optimal development that enables their resilience. Equity of resources within LSEN schools and diverse contexts will not be attained any time soon, and the reality may be that equity may not be possible at all. This difficulty calls for research to uncover resilience-promoting resources in different contexts and LSEN schools in South Africa. Prior studies have shown that in different contexts, even with fewer resources, resilience is prevalent, indicating that everyone is capable of resilience regardless of the limiting circumstances they find themselves in. The communities and LSEN schools may be less resourced; however, the little resources the contexts have can be used meaningfully to enable the resilience of learners with SLD.

It is, thus, important that learners with SLD should be capacitated with the ability to navigate their environments for resilience enablers. This study calls on stakeholders (such as parents, school personnel and community members) to capacitate learners with SLD with skills to navigate their contexts and search for resources that promote resilience. These skills include planning tasks to identify resources and interviewing community members about any available resources. This study emphasises the individual’s capacity to navigate resilience-enabling resources, which could be integrated into school lessons. The curriculum at school needs to consider empowering learners with SLD with life and resilience skills, such as effective ways to handle unfavourable life circumstances. School tasks could include community mapping and the purpose will be to explore available and accessible services and structures in their respective contexts. Social-ecology stakeholders, such as parents, family members, peers, teachers, neighbours and community members, should collaborate to build learners’ capacity to navigate their contexts. One of the limitations observed in this study was that some of the learners in Soweto struggled with reading the questionnaire and presented with English language difficulties. The limitation was addressed by reading for them and on-the-spot translation of the CYRM-28 into their mother tongue to ensure accurate comprehension of the items. The reliability of the results could have been compromised. Further research on the topic can be considered using a qualitative research approach. In addition, similar research can be replicated in other contexts in South African and LSEN schools outside the Gauteng province.
